# Research Progress of Electrical Stimulation in Ischemic Heart Disease

**DOI:** 10.3389/fcvm.2021.761877

**Published:** 2021-11-03

**Authors:** Ying Zhao, Pengyu Wang, Zhe Chen, Manman Li, Dengfeng Zhang, Liming Yang, Hong Li

**Affiliations:** ^1^Department of Pathophysiology, School of Basic Medical Sciences, Harbin Medical University, Harbin, China; ^2^Department of Infectious Diseases, Beidahuang Group General Hospital, Harbin, China; ^3^Department of Pathophysiology, Harbin Medical University-Daqing, Daqing, China

**Keywords:** electrical stimulation, ischemic heart disease, angiogenesis, apoptosis, autophagy, inflammation, oxidative stress

## Abstract

Ischemic heart disease (IHD) is a considerable health burden worldwide with high mortality and morbidity. Treatments for IHD are mainly focused on decreasing oxygen demand or increasing myocardial oxygen supply, including pharmacological, interventional, and surgical treatment, but there are also some limitations. Therefore, it is important to find a simple, effective, and economical treatment. As non-invasive and safe physiotherapy, electrical stimulation (ES) has a promising application in the treatment of IHD. Current studies suggest that ES can affect the occurrence and development of IHD by promoting angiogenesis, regulating autophagy and apoptosis, inhibiting the inflammatory response and oxidative stress. In this review, we focus predominantly on the mechanism of ES and the current progress of ES therapy in IHD, furthermore, give a brief introduction to the forms of ES in clinical application.

## Introduction

Cardiovascular disease has been a serious threat to human life for a long time and its incidence is increasing (1). Coronary artery disease (CAD), also known as ischemic heart disease (IHD), is the main cause of heart failure, which seriously endangers the health of people and puts a huge burden on health care resources all over the world. In China, more than half of patients with heart failure developed from IHD (2). Therefore, it is a common problem for clinicians and researchers to explore the pathogenesis of IHD and carry out effective prevention and treatment. According to clinical studies, the onset of IHD is a long-term and complex process, which mainly involves pathophysiological changes such as coronary artery stenosis, coronary artery thrombosis and embolism, vasospasm, microcirculation disorder, inflammation, endothelial dysfunction, apoptosis, and autophagy cascading reactive activation, etc (3). It is mainly caused by the spread of severe coronary artery stenosis or complete blockage of chronic coronary arteries, resulting in an imbalance between the oxygen supply and oxygen demand of the myocardial cells, impaired myocardial energy metabolism leading to autophagy and ischemia-reperfusion injury, which then leads to a syndrome manifesting as heart enlargement, heart failure, arrhythmias, and other symptoms (4)_._ In recent years, with the continuous development of molecular biology, people have a deeper knowledge and understanding of the occurrence and development of IHD. Furthermore, treatments for IHD are mainly focused on decreasing oxygen demand or increasing myocardial oxygen supply, including pharmacological, interventional, and surgical treatment, but there are also some limitations (5). Thus, it is important to find a simple, effective, and economical treatment.

**Figure 1:**
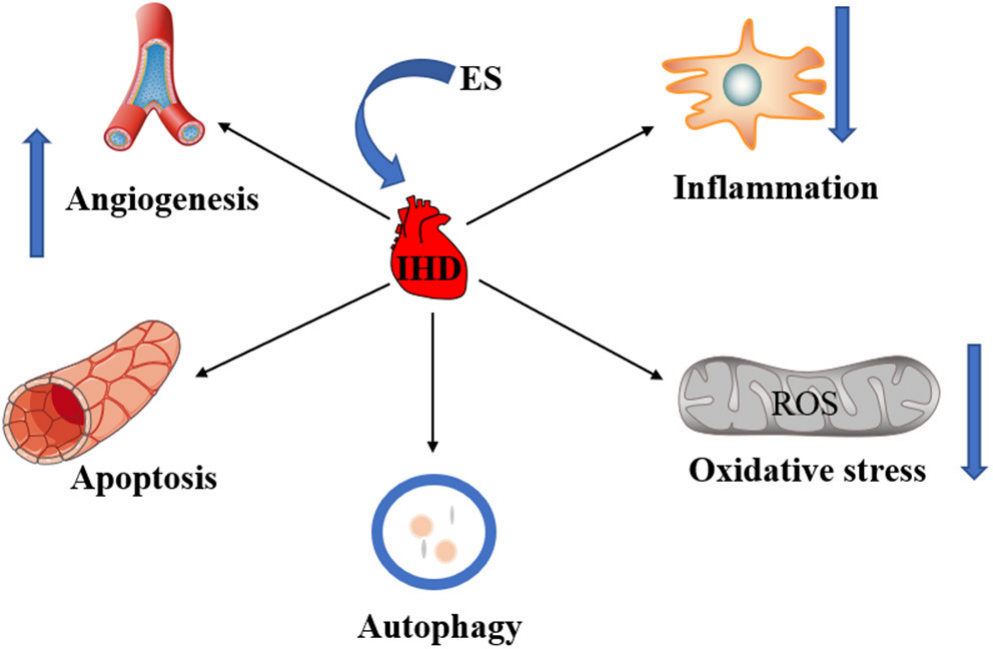
The role of ES in IHD. ES can treat IHD by promoting angiogenesis. ES can treat IHD by regulating apoptosis and autophagy. The inhibition of inflammation and oxidative stress by ES is also critical to the treatment of IHD.

The bioelectric phenomenon is one of the most basic life activities, almost every physiological process in the human body is related to bioelectricity, such as heart beating, muscle contraction, brain thinking. As early as the 19th century, scientists had discovered that exogenous ES of the human cortex led to behavioral changes (6). Since then, a large number of studies have demonstrated that electric fields can cause physiological changes in cells. ES therapy is a physical therapy method that uses electrical currents to cause changes in the morphology and function of cells or tissues through different pathways. In recent years, with the continuous development of control technology of ES and the deepening of research on different types of stimulation, the application of ES in IHD has received more and more attention. As non-invasive and safe physiotherapy, ES has been widely used in the rehabilitation of many diseases such as neuromuscular injuries (7). New advances in IHD are achieved through the application of different types of ES to influence cell proliferation and differentiation. Current studies suggest that ES can affect the occurrence and progress of IHD by promoting angiogenesis, regulating autophagy and apoptosis, inhibiting the inflammatory response and oxidative stress (Figure 1). Here, the role of ES in IHD was reviewed to further analyze the mechanism of ES and provide new ideas for the treatment of clinical IHD.

## Overview of Electrical Stimulation

In more than 300 BC, Aristotle observed that electric rays paralyzed animals in the water by striking them with shock as they hunt. It was only after the 18th century, when the laws of electricity were discovered, that people gradually realized the nature of the biological discharge. With the invention of the electric motor and the battery, people began to figure out electricity. Then many scientists proved the existence of bioelectricity. With the invention of the galvanometer, people could measure bioelectricity directly. Subsequently, bioelectricity was studied, a membrane theory was developed and the transmembrane potential of cells was recorded. At this time, the research is not lotted to the phenomenon and the mechanism of occurrence. With the deepening of the research, people began to try to observe the biological electrical phenomenon of the body. Its principle was to use the electric excitability of nerve cells to stimulate the specific sensory nerve with electricity, and produce nerve impulses to produce the corresponding immune and humoral response in the area dominated by it, to play the role of regulating the body (8). ES can promote biomolecules to cross biofilms through ion osmosis, or interact with membrane proteins, cytoskeleton, and intracellular organelles (9, 10). ES therapy is a kind of physical therapy that uses electrical currents to cause changes in the morphology and function of cells or tissues through different pathways (11). Based on these remarkable advantages, it is now beginning to be widely used in the biomedical field (11). ES has become an active area of research in nerve, cardiac and skeletal muscle engineering. In tissue engineering and regenerative medicine, ES can directionally accelerate neuronal axon growth and embryonic stem cell differentiation into neuron (12). In addition, ES can stimulate the growth of nerve axons and increases cell alignment (13, 14). ES is already used in tissue engineering to improve the conduction and contractile properties of heart structure (15). Recent reports have shown that macrophages play a role in electrical conduction beyond the atrioventricular node, including atrial fibrillation and ischemic ventricular arrhythmias (16). IHD causes abnormalities in cardiac conduction. Stimulation of the carotid sinus has been shown to affect blood flow in areas of myocardial ischemia to relieve angina. Whether ES of the carotid sinus nerve improves conduction abnormalities in IHD *via* sinus node conduction needs to be further investigated. ES can promote the proliferation of osteoblasts, regulate the local endocrine environment, participate in osteogenic differentiation, and promote bone formation in bone tissue engineering (17). Ischemia-reperfusion injury is common pathological damage in the clinic of IHD. Currently, experts are trying to use ES to alleviate myocardial ischemia-reperfusion injury (18). Ninety percentage of IHD is caused by the narrowing of the coronary lumen caused by atherosclerosis, resulting in problems of myocardial ischemia and hypoxia. Our previous study found that ES could reduce macrophage inflammatory response, reduce lipid accumulation in foam cells, and reduce AS plaque area (19). As a new technique, ES has been widely used in the treatment of IHD. Although its specific mechanism remains unclear, it provides a new direction for the treatment of IHD.

### Types of Electrical Stimulation

In recent years, with the continuous development of ES control technology, many different types of ES had been applied in biomedicine and achieved good efficacy. Depending on the output signal, ES can be in the form of a single-phase (DC) or two-phase (AC), sinusoidal, sawtooth, or square-wave signal, pulse, pulse burst, or continuous pulse. Of these, the most basic is to apply a direct current voltage (20–25). ES can be divided into excitatory electrical stimulation and non-excitatory electrical stimulation according to whether the action potential is generated after ES. Current studies have shown that non-excitatory electrical stimulation on the left ventricular wall of rats can reduce cardiac ischemia-reperfusion injury by reducing the expression of calcitonin gene-related peptide (CGRP) (26). With the development of ES, so far, there have been many forms. First of all, studies have shown that percutaneous ES of the auricular branch of the vagus nerve has been applied to treat epilepsy and depression. Both animal experiments and clinical practice have proved their safety and effectiveness. Therefore, ES of the auricular branch of the vagus nerve would be a new non-invasive method to optimize cardiac autonomic tone in the treatment of ischemic heart failure (27–29). Another particular type of ES is electroacupuncture (EA). EA is a special form of ES, that is nervous electrical stimulation combined with acupuncture therapy and physical therapy, refers to the insertion of traditional Chinese medicine, based on pulse generator on the acupuncture needle passed close to the trace of human bioelectricity current wave, based on the original needle stimulates, attaches a certain amplitude of the continuous wave, discontinuous wave to stimulate acupuncture points, sustained, stable and accurate for the body, can be accused of stimulus, the electrophysiological effect of therapeutic effect (30). EA is easy to perform and avoids many of the adverse effects of drug pre-treatment. EA preconditioning, as an effective method of myocardial protection, can activate the endogenous protective mechanism of the body, mobilize the potential of the body, and improve the ability of the myocardium to adapt to the stress response. It has the advantage of adapting to a wide population and has a broad clinical application prospect. In recent years, animal experiments have shown that EA affects the whole process of autophagy flux by regulating the expression of the ULK1 complex, Beclin1, LC3, p62, and other autophagy-related markers. Further studies have shown that EA therapy may play a neuroprotective role in cerebral ischemia/reperfusion injury by regulating autophagy through the PI3K-Akt-mTOR signaling pathway (31). Current studies have shown that the locations of ES are also different, including the vagus nerve, spinal cord, cerebellar parietal nucleus, as well as the left cervicothoracic joint, sinoatrial node, and Neiguan acupoint (27, 28, 30). Scientists are trying to find areas where ES is most effective and least harmful to provide better theoretical and technical support for treating diseases with ES.

## Roles of es in IHD

### Angiogenesis

IHD is a condition in which the heart is affected by myocardial ischemia caused by narrowing or occlusion of the coronary arteries. The establishment of collateral coronary artery and its degree of development can reduce or prevent myocardial ischemia or necrosis and improve cardiac function. The body can promote the establishment of coronary collateral circulation through capillary angiogenesis. The role of angiogenesis in cardiovascular disease is still an important and unsolved problem, and there are different views on it in the medical community. Angiogenic cytokines have been widely considered to be useful in the treatment of IHD. As a new modulator, ES has been used in many ischemia models. ES can regulate angiogenesis by affecting cytokines and their receptors. However, the specific mechanism remains unclear and further studies are needed, but a large number of experiments have shown that ES has great potential in inducing angiogenesis and improving organ function.

#### Angiogenesis and IHD

IHD is a global public health problem with high morbidity and mortality. The main pathophysiological process of IHD is atherosclerosis. Coronary artery wall atherosclerosis causes stenosis of the vascular lumen, obstruction of coronary circulation, and insufficient blood supply to the myocardium, leading to myocardial ischemia, myocardial infarction, and other IHD (32). Importantly, restoring the blood supply is key to the successful treatment of IHD. Current treatments for IHD include drug therapy and surgical interventions that reduce the risk of cardiovascular events in IHD by dilating blood vessels to increase blood flow to damaged tissues. Angiogenesis is a novel therapeutic approach that can alleviate the symptoms of IHD (33). Angiogenesis is the development of new blood vessels from existing capillaries or post-capillary veins. Angiogenesis is an essential event involved in various physiological and pathological processes such as development, wound healing, and tumor growth (34). It involves the degradation of the vascular basement membrane, activation, proliferation, and migration of vascular endothelial cells during the activation phase; the reconstruction of blood vessels and vascular networks is a complex process involving multiple molecules and cells. Angiogenesis is a complex process of coordination between proangiogenic factors such as vascular endothelial growth factors (VEGF), fibroblast growth factors (FGF), and antiangiogenic factors such as thrombospondin-1, angiostatin, endostatin (35). VEGF (Vascular endothelial growth factor) is a subfamily of growth factors that stimulate the growth of new blood vessels and is an important signaling protein involved in angiogenesis and angiogenesis (36). Angiogenesis can promote the establishment of collateral circulation in ischemic peripheral tissues. The formation and opening of coronary collateral circulation can alleviate myocardial ischemia, prevent cell necrosis, prevent and delay the formation of ischemic cardiomyopathy, and improve the clinical symptoms and prognosis of patients. Studies have shown that neovascularization can effectively restore coronary blood perfusion and promote myocardial regeneration (37).

#### ES Can Promote Angiogenesis for IHD

**Figure 2:**
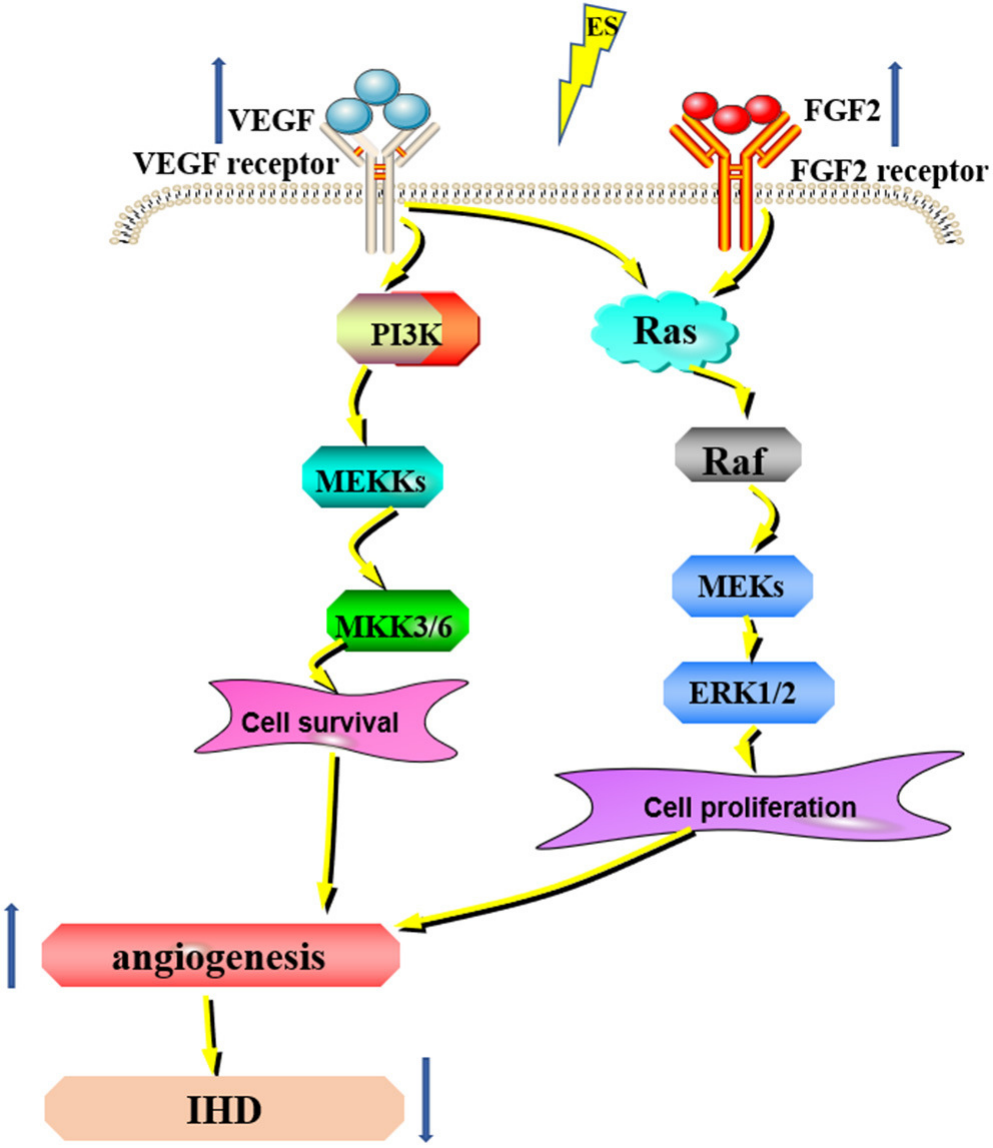
ES can promote angiogenesis for IHD. ES can increase the expression of VEGF and FGF2 and promote angiogenesis through the MAPK/ERK signaling pathway, thus treating IHD.

Recently, subthreshold electrical stimulation is widely used in wound healing, fracture repair, and other clinical diseases due to its potential therapeutic ability. Given the experimental evidence that ES can promote wound healing by inducing the release of angiogenic factors and reducing the duration of the inflammatory period (38). The enhancement of angiogenesis by ES is considered to be a new chapter in the search for new methods to treat ischemia (39). Additionally, studies have shown that ES facilitates the angiogenesis of human umbilical vein endothelial cells through MAPK/ERK signaling pathway by stimulating FGF2 secretion (40) (Figure 2). Moreover, it has been shown that the expression of the VEGF gene is upregulated in electrically stimulated rat skeletal muscle (41). ES directly induces the proangiogenic response of vascular endothelial cells through VEGF receptors (42, 43) (Figure 2). Twenty-five hertz and 50Hz low voltage ES can promote the expression of VEGF in the ischemic myocardium and lead to capillary angiogenesis (44, 45). The specific effects of ES on the downstream pathways of VEGF and FGF2 still need further study. Therefore, ES can promote angiogenesis in the ischemic myocardium, but the specific mechanism is still unclear and needs further study. Taken together, in the future, ES will be expected to be a simple and practical new method to promote angiogenesis in the ischemic myocardium.

### Apoptosis

Coronary arteriosclerosis is the main cause of IHD. When the insufficient blood supply to the coronary artery of the heart fails to meet the energy needs of the myocardium, the myocardial tissue will accumulate toxic metabolites due to hypoxia and metabolic disorders, which will cause ischemic injury, and lead to myocardial death if it continues to develop. Apoptosis is an important mode of death of ischemic cardiomyocytes. ES is a new physical method to treat IHD by regulating apoptosis, the specific mechanism is not clear, there are some theories and hypotheses that need to be further confirmed, but the study of ES for the treatment of cardiovascular disease provides new thinking and method.

#### Apoptosis and IHD

Apoptosis is a form of programmed cell death that requires protein synthesis and a highly regulated process of cellular self-destruction by specific cellular signals and proteins (46). Apoptosis is a process strictly controlled by multiple genes. These genes are very conserved among species, such as the Bcl-2 family, caspase family, oncogene P53, etc. With the development of molecular biology techniques, there is considerable understanding of the process of apoptosis in a variety of cells, but the exact mechanism of apoptosis has not been fully understood up to now. And the disorder of the apoptotic process may have a direct or indirect relationship with the occurrence of many diseases. Such as tumors, autoimmune diseases, etc. Many factors can induce apoptosis, such as radiation, drugs, etc (47–50). IHD is a clinical syndrome that results in myocardial ischemia and hypoxia, apoptosis, and myocardial fibrosis caused by coronary atherosclerosis. Several recent studies have also confirmed the important role of apoptosis in IHD. Excessive apoptosis can promote myocardial cell death thus promoting myocardial ischemia, ischemia/reperfusion (I/R) injury, and post-ischemic cardiac remodeling in IHD (51, 52). In general, it is very important to find a new way to regulate apoptosis to contribute to the treatment of IHD and to provide a new therapeutic strategy.

#### ES Treats IHD by Regulating Apoptosis

**Figure 3:**
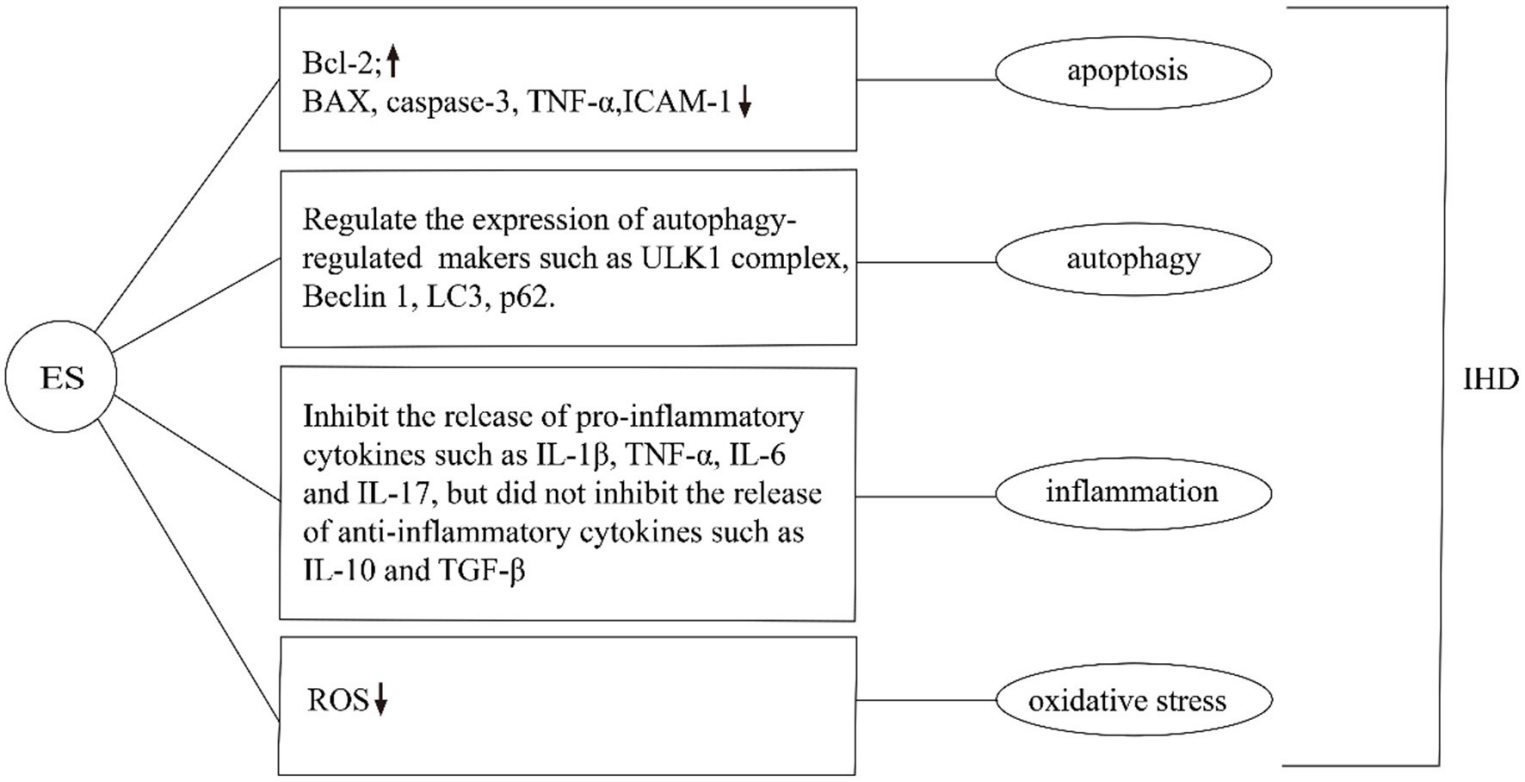
Effects of ES on IHD in apoptosis, autophagy, inflammation, and oxidative stress. ES can regulate apoptosis by changing the expression of intracellular proteins. For example, Increase the expression of Bcl-2, inhibit the expression of Bax, Caspase-3, TNF-α, ICAM-1. ES can regulate the expression of autophagy-related markers such as ULK1 complex, Beclin1, LC3, and p62. Moreover, it can inhibit the release of pro-inflammatory cytokines such as IL-1β, TNF-α, IL-6, and IL-17, but did not inhibit the release of anti-inflammatory cytokines such as IL-10 and TGF-β. Additionally, ES can reduce ROS and inhibit oxidative stress in the treatment of IHD.

Subthreshold electrical stimulation is a method used to manipulate cells to induce changes in various cellular processes, such as apoptosis and cell proliferation (53–55). Controversially, it has been shown that ES can both inhibit or induce apoptosis and promote cell proliferation. It has been shown that ES induces apoptosis by altering the permeability of the cell membrane and forming small pores in the cell membrane (electroporation) (56). In turn, it activates the apoptosis-related signal pathways and regulates apoptosis by altering the expression of p53 and Bcl-2 proteins (57) (Figure 3). ES can reduce apoptosis by altering intracellular protein expression and activating multiple signaling pathways (such as MAP kinase pathway) (58). ES to reduce apoptosis can also be used in the treatment of IHD. For instance, subthreshold electrical stimulation may reduce the apoptosis of rat ischemic cardiomyocytes by up-regulating the gene expression of Bcl-2 and down-regulating the gene expression of Bax, but the exact mechanism is unclear (59). In addition, other studies have shown that 25 Hz subthreshold electrical stimulation can reduce the apoptosis of ischemic cardiomyocytes in rats, and the mechanism may be related to the down-regulation of caspase-3 expression. Effective stimulation of Neiguan acupoints with EA pretreatment can reduce the production and level of TNF-α and intercellular adhesion factor-1 (ICAM-1) during myocardial ischemia-reperfusion injury, thereby reducing apoptosis of cardiomyocytes and tissue cells and suppressing the degree of the inflammatory response to effectively regulate heart rate and increase cardiac blood output in patients after coronary artery bypass grafting for coronary artery disease, resulting in a decrease in cardiac troponin T concentration and contributing to the maintenance and improvement of cardiac function. Subthreshold electrical stimulation in both ischemic and non-ischemic areas can reduce apoptosis of ischemic cardiomyocytes in rats (60). The effect of EA on the expression of caspase genes and similar sequences in cardiac myocytes was significant, and it could protect myocytes in the ischemic area during ischemia-reperfusion by slowing down the apoptosis process of cardiac myocytes. The mechanism of the effect of EA stimulation on the prevention of myocardial hypertrophy in rats is related to the modulation of the ERK signaling pathway, and its effect on the prevention of myocardial hypertrophy may be through the inhibition of the neuroendocrine system angiotensin II and endothelin to regulate the ERK signaling pathway to exert anti-apoptotic effects on cardiomyocytes (61). However, it is noteworthy that the mechanism by which ES achieves this goal is unclear. Further studies by scientists are needed to provide a new direction for ES to regulate apoptosis.

### Autophagy

Autophagy generally exists in the normal physiological and pathological processes of cells. Appropriate autophagy can be used to maintain homeostasis, but insufficient and excessive autophagy can lead to related diseases. IHD is involved in the complex regulation of autophagy in myocardial hypertrophy, myocardial fibrosis, and the efficacy after reperfusion therapy, both in the ischemic stage and in the later stage. At present, the role of autophagy in IHD has increasingly become the focus of research. The regulation of autophagy by ES has been studied in a variety of diseases, but the study in IHD is still in the preliminary stage, and the specific mechanism is not clear yet, which needs further study to confirm. Studies on the regulation of autophagy by ES will provide a new direction for the treatment of IHD.

#### Autophagy and IHD

Autophagy is a general term for intracellular lysosomal degradation substances that are considered essential for the maintenance of normal cardiac structure and function. It is also associated with several cardiac diseases, especially myocardial ischemia/reperfusion (I/R) injury (62). Autophagy plays an important role in maintaining normal cell homeostasis and energy metabolism balance and is a necessary condition for regulating cardiovascular function (63). There are three main forms of autophagy: microautophagy, autophagy with molecular chaperones, and macrophage, among which macrophage is the most common and the most thoroughly studied. There are several stages in the progression of macroautophagy, including autophagosome initiation, vesicle nucleation, autophagosome expansion and maturation, and autophagosome fusion and degradation (64). There is growing evidence for a regulatory role of autophagy in IHD. Recent studies have demonstrated its dual role in IHD. Moderate autophagy is thought to be cardioprotective, while excessive autophagy exacerbates cardiomyocyte death (65). During the myocardial ischemic phase, autophagy degrades nonfunctional cytoplasmic proteins to provide critical nutrients for key vital activities, thereby inhibiting apoptosis and necrosis. However, autophagy may negatively affect the heart during the reperfusion phase. During the ischemic phase, mTOR acts through the AMPK/mTOR and phosphatidylinositol 3-kinase/Akt/mTOR pathways, whereas during the reperfusion phase, Beclin1 is upregulated (66). From what has been discussed above, the regulation of autophagy is helpful for the treatment of IHD.

#### ES Can Mitigate Cardiomyocyte Injury by Regulating Autophagy

Latterly, Studies have shown that subthreshold electrical stimulation plays an important role in the whole process of autophagy, including the initiation of autophagy, vesicle nucleation, expansion, and maturation of autophagic vesicles, and fusion and degradation of autophagic lysosomes (67–69). Currently, the results of animal experiments have shown that ES affects the whole process of autophagic flux by regulating the expression of autophagy-related markers such as ULK1 complex, Beclin1, LC3, and p62 (Figure 3). For instance, after ES was applied to skeletal muscle, the activities of autophagy autophagy-related proteins, were significantly increased, and the expression of the ubiquitin-ligase gene of proteasome and mRNA of autophagy-related genes were significantly up-regulated, suggesting that the level of autophagy in skeletal muscle was increased, and the mechanism was related to the regulation of adenylate activated protein kinase/ULK1-mediated signaling pathway (70). Another study suggests that ES treatment may regulate autophagy through the PI3K-AKT-mTOR signaling pathway, thus exerting neuroprotective effects in cerebral ischemia/reperfusion injury. The modulation of cerebral ischemia/reperfusion injury by autophagy depends on the duration of ischemia/reperfusion and parameters such as the selected acupuncture point, current intensity, waveform, and duration (31). EA may moderately regulate the autophagy level during MIRI by down-regulating the expression of LC3II and Beclin-1 proteins. EA pretreatment can effectively improve the pathological changes of the myocardium and reduce the damage of myocardium in rats with MIRI (71). ES can not only promote autophagy but also inhibit over-autophagy through different intensification of ES. The hippocampus of rats treated with ES can inhibit over-autophagy by activating the mammalian target pathway of rapamycin, thus exerting a brain-protective effect (72). The above findings indicate that ES is of potential significance in IHD treatment. However, the precise mechanisms involved remain obscure, and therefore more in-depth exploration and more adequate validation are needed to elucidate the clinical value of ES in IHD.

### Inflammation

Inflammation is one of the research hotspots of the risk factors of IHD in recent years. When the body is in an inflammatory state, it can promote the occurrence of acute cardiac events of coronary heart disease, and affect the function and prognosis of the ischemic heart. Inflammation is crucial in the pathogenesis of IHD, but there is still a lack of a complete signaling network, and the role of inflammation in the pathogenesis remains unclear. As a new intervention means, ES has been shown in numerous studies to regulate inflammatory pathways and inhibit the secretion of inflammatory mediators. The regulation of ES on inflammation in IHD has also been studied, suggesting that ES can be used as a potential new method to inhibit inflammation in the treatment of IHD, but the specific mechanism remains to be further studied.

#### Inflammation and IHD

Inflammation plays an important role in atherosclerosis and cardiovascular disease. A large amount of research is currently being performed to demonstrate that inflammation is closely related to the development of atherosclerosis and coronary artery disease (73–75). Atherosclerosis is associated with activation of inflammatory processes and an increase in systemic pro-inflammatory molecules such as interleukin 1 beta (IL-1β), interleukin 6 (IL-6), tumor necrosis factor (TNF), and c-reactive protein (76). It has been shown that activation of NOD-like receptor family protein 3 (NLRP3) inflammasome leads to increased inflammation in ischemic myocardial tissue, which further worsens cardiac dysfunction and becomes a key component of the post-ischemic inflammatory response to myocardial ischemia as well as ischemic tissue repair (77). Modulating the activation of NLRP3 inflammatory vesicles can balance the inflammatory homeostasis of ischemic myocardial tissue and facilitate the repair and remodeling of ischemic myocardial tissue after injury. Numerous animal experiments have shown that NLRP3 expression is elevated in ischemic myocardial tissue and the inflammatory response is exacerbated. Conversely, the absence of NLRP3 inflammatory vesicle components attenuates the inflammatory response and promotes cardioprotective effects (78). In conclusion, it is easy for us to conceive that suppressing inflammation may represent a promising IHD treatment strategy.

#### ES Can Inhibit Inflammation to Treat IHD

**Figure 4:**
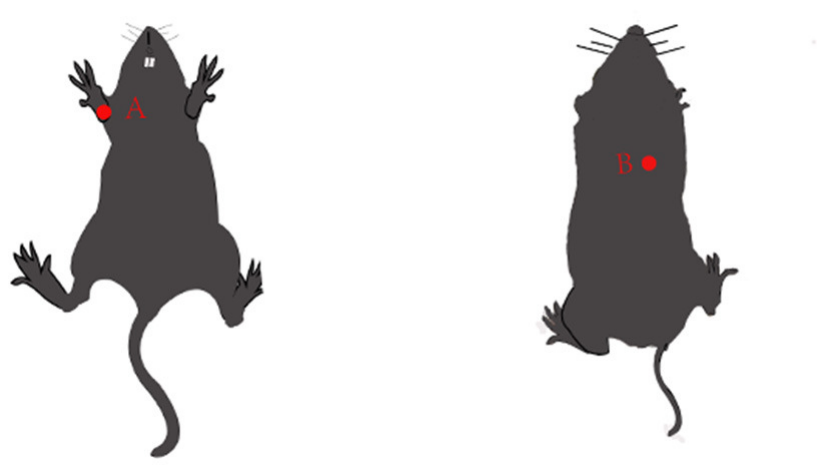
Pattern diagram of mouse acupuncture points. **(A)** Neiguan. Neiguan point was located in the medial side of the forelimb, between the ulnar and radial joints, saving 3 mm from the wrist pass; **(B)** Xinyu. Xinyu acupoint in the back, the fifth thoracic spinous process, besides 1.5 inches. That's about an inch and a half on each side of the fifth vertebra.

Excitatory electrical stimulation can modulate the cholinergic anti-inflammatory pathway to inhibit the release of inflammatory mediators, thereby slowing the onset and progression of various inflammation-related diseases (79). The cholinergic anti-inflammatory pathway composed of the vagus nerve and its transmitter acetylcholine plays an important role in the regulation of inflammatory response. When the body is injured, the excitability of the vagus nerve increases, which promotes the release of acetylcholine from the peripheral nerve endings. It can inhibit the release of pro-inflammatory cytokines such as IL-1β, TNF-α, IL-6, and IL-17, but did not inhibit the release of anti-inflammatory cytokines such as IL-10 and TGF-β (80) (Figure 3). ES can also alter the function of inflammatory cells at the molecular level, thereby inhibiting the release of inflammatory mediators, without affecting the number of immune cells (81). The anti-inflammatory effect of electrical stimulation has been applied in the clinical treatment of various inflammation-related diseases. It has been shown that transcutaneous electrical stimulation of the vagus nerve can promote wound healing by suppressing the inflammatory response (82). ES can be used to treat ocular diseases by enhancing the neurotrophic potential of Muller cells and inhibiting the pro-inflammatory effect of microglial cells, thus improving the denaturing degree of photosensors (83, 84). ES has been widely used to regulate neuronal activity and restore some visual function (85). Additionally, diagenesis of the lumbar intervertebral disc is the main cause of lower back pain. Inflammatory factors were released near nerve roots, affected nerve roots, and caused pain. ES can reduce the activity of extracellular matrix modifier enzyme and matrix metalloproteinase-1, inhibit the secretion of inflammatory cytokines, and achieve the effect of pain relief (86). The stimulation of the apical splenic nerve by ES can reduce the inflammatory response and clinical symptoms in a mouse model of rheumatoid arthritis (87). Previous studies by our group have shown that ES downregulated the expression of the NLRP3 inflammasome, ASC and caspase-1, and inhibited the release of IL-1β and IL-18, suppressed the AS pyroptosis-mediated inflammation response (19). ES can inhibit the inflammatory response and experimental myocardial protective effect by reducing the level of inflammatory protein expression in myocardial tissue. EA at “Neiguan” and “Xinyu” points significantly reduced serum IL-1β content and NF-κB p65 protein expression level in rats with acute myocardial ischemia-reperfusion injury, and increased IL-10 content in myocardial tissues (88) (Figure 4). In conclusion, the above studies show that from bench to bedside, ES can inhibit inflammation to treat IHD. However, the specific mechanism is still unclear and needs further study.

### Oxidative Stress

In the course of treatment, reperfusion may cause myocardial ischemia-reperfusion injury due to aggravation of oxidative stress state. Oxidative stress induced by ischemia acts on heart cells, and through different molecular mechanisms, can produce a series of pathological reactions, resulting in myocardial damage and impaired cardiac function. ES has the function of scavenging oxygen free radicals and inhibiting the damage of oxidative stress response to the central nervous system and heart. However, the specific mechanism remains to be further studied.

#### Oxidative Stress and IHD

It is well known that the occurrence, progression, metastasis, and other processes of IHD involve complex molecular mechanisms, and the current understanding of them is incomplete, which leads to a low cure rate of IHD. It is recognized that oxidative stress is closely related to coronary artery disease. In the pathological process, oxidative stress is induced, and its main characteristic is the elevated level of reactive oxygen species (ROS). As a major mediator, ROS not only deteriorates cell physiological functions and causes cell damage through its oxidative activity, but also participates in cell signal transduction and regulates disease progression, namely REDOX signal (89, 90). In IHD, excessive production of (ROS) leads to dysfunction of endothelial cells and smooth muscle, resulting in an imbalance between antioxidant capacity and oxidants (91). During myocardial ischemia, the production of free radicals and the activity of antioxidant enzymes is increased, which can lead to severe damage of myocardial cells to apoptosis. The activities of superoxide dismutase (SOD) and glutathione peroxidase (GSH-Px) in myocardial cells of the ischemic area were increased. Taken together, these findings suggest that reducing oxidative stress may serve as a novel treatment for IHD.

#### ES Can Reduce Oxidative Stress and Protect Cardiomyocytes

ES can be used to protect cells and tissues from damage caused by ROS (92). Intraoperative ES reduced oxidative stress and up-regulated the level of stimulating diaphragmatic autophagy (93). Studies have shown that electroacupuncture pretreatment and stimulation of Neiguan acupoint can significantly improve the enzyme activity of SOD, GSH-Px, and other endogenous oxidation free radical scavenging systems, inhibit the lipid peroxidation reaction of the myocardial cell membrane, reduce the content of serum MDA, and play a protective role in myocardium (94). Other studies have found that EA preconditioning has an obvious protective effect on the myocardium after reperfusion injury, can improve arrhythmia score, downregulate CK-MB level, regulate ROS production, and reduce the expression levels of CYT-C, Caspase-9, and Caspase-3 genes. Its myocardial protective effect may be based on the inhibition of ROS-mediated apoptosis of cardiomyocytes (95) (Figure 3). Our previous experiments have shown that ES can reduce VBP-induced oxidative stress in macrophages, thereby inhibiting caspase-1-dependent cell apoptosis. Meanwhile, ES can increase the expression of SIRT3 and improve VBP-induced autophagy of macrophages, thereby reducing oxidative stress (19). Overall, these researches suggest that the inhibition of oxidative stress by ES has the potential to be used as a new treatment for IHD.

### Other Mechanisms

Current studies have shown that ES can also protect cardiomyocytes through other mechanisms to treat IHD. ES can inhibit intracellular calcium overload and maintain calcium homeostasis to protect the myocardium. EA stimulation of Neiguan acupoint can promote the gene expression of Ca^2+^-ATPase mRNA in ischemia-reperfusion myocardial cells, which can improve the effect of alleviating myocardial injury and protecting myocardial function (96). ES of the left cervicothoracic ganglion and the Neiguan acupoint-Jiangrong acupoint can down-regulate the expression of norepinephrine and catecholamine in the rat and rabbit myocardial ischemia models, respectively, and increase the blood flow in the infarction area, thus alleviating the myocardial ischemia-reperfusion injury (97, 98). ES of Neiguan acupoint can play a cardiac protective role by activating opioid receptors in the medulla oblongata and regulating the concentration of cAMP and cGMP in cardiomyocytes (99). Overall, ES is involved in the regulation of many cellular signaling pathways and plays a significant role in the occurrence, development, and clinical manifestations of IHD, indicating that ES is a potential way for IHD therapy.

## Conclusion

At present, as a new intervention means, ES has the characteristics of non-invasive, safe, and fewer side effects, etc., and has been paid more and more attention in IHD. There is growing evidence that subthreshold electrical stimulation can promote angiogenesis, modulate autophagy and apoptosis, inhibit oxidative stress and excitatory electrical stimulation can inhibit inflammation via cholinergic anti-inflammatory pathways in IHD. However, the study of ES in the treatment of IHD should be in its infancy. Although ES has been used in the clinical treatment of nerve, bone, gynecology, and other diseases, some ES sites are special, and it is dangerous to directly use in clinical practice, which hinders its clinical application. Therefore, an in-depth understanding of the mechanism of ES in the treatment of IHD, as well as the search for appropriate parameters and appropriate implementation methods and locations that can effectively play a role, will help to provide better scientific data for clinical application and increase the feasibility of a clinical application. In conclusion, further in-depth mechanism research is the future research direction of ES. We believe that ES will certainly provide new ideas for treating IHD.

## Data Availability Statement

The original contributions presented in the study are included in the article/supplementary material, further inquiries can be directed to the corresponding authors.

## Author Contributions

YZ, HL, and LY designed and wrote the manuscript. YZ, PW, ZC, ML, DZ, LY, and HL edited the manuscript. All authors contributed to the article and approved the submitted version.

## Funding

This review was funded by the National Natural Science Foundation of China (91939104, 82070465, 82170469); Heilongjiang Province Science Foundation for Distinguished Young Scholars (JQ2021H001); General project of Heilongjiang Provincial Health Commission (2020-075); Construction Project of Scientific Research and Innovation Team of Harbin Medical University-Daqing (HD-CXTD-202001); Key Discipline Construction Project of Harbin Medical University-Daqing (HD-ZDXK-202001).

## Conflict of Interest

The authors declare that the research was conducted in the absence of any commercial or financial relationships that could be construed as a potential conflict of interest.

## Publisher's Note

All claims expressed in this article are solely those of the authors and do not necessarily represent those of their affiliated organizations, or those of the publisher, the editors and the reviewers. Any product that may be evaluated in this article, or claim that may be made by its manufacturer, is not guaranteed or endorsed by the publisher.
